# The functional role of L-fucose on dendritic cell function and polarization

**DOI:** 10.3389/fimmu.2024.1353570

**Published:** 2024-04-05

**Authors:** Chase Burton, Amirreza Bitaraf, Kara Snyder, Chaomei Zhang, Sean J. Yoder, Dorina Avram, Dongliang Du, Xiaoqing Yu, Eric K. Lau

**Affiliations:** ^1^ Department of Immunology, H. Lee Moffitt Cancer Center & Research Institute, Tampa, FL, United States; ^2^ Cancer Biology Ph.D. Program, University of South Florida, Tampa, FL, United States; ^3^ Immunology Program, H. Lee Moffitt Cancer Center & Research Institute, Tampa, FL, United States; ^4^ Molecular Medicine Program, H. Lee Moffitt Cancer Center & Research Institute, Tampa, FL, United States; ^5^ Department of Tumor Microenvironment and Metastasis, H. Lee Moffitt Cancer Center & Research Institute, Tampa, FL, United States; ^6^ Department of Molecular Medicine, University of South Florida, Tampa, FL, United States; ^7^ Molecular Genomics Core, H. Lee Moffitt Cancer Center & Research Institute, Tampa, FL, United States; ^8^ Department of Biostatistics and Bioinformatics, H. Lee Moffitt Cancer Center & Research Institute, Tampa, FL, United States

**Keywords:** dendritic cells, L-fucose, myeloid cells, antigen presentation, tumor immune microenvironment

## Abstract

Despite significant advances in the development and refinement of immunotherapies administered to combat cancer over the past decades, a number of barriers continue to limit their efficacy. One significant clinical barrier is the inability to mount initial immune responses towards the tumor. As dendritic cells are central initiators of immune responses in the body, the elucidation of mechanisms that can be therapeutically leveraged to enhance their functions to drive anti-tumor immune responses is urgently needed. Here, we report that the dietary sugar L-fucose can be used to enhance the immunostimulatory activity of dendritic cells (DCs). L-fucose polarizes immature myeloid cells towards specific DC subsets, specifically cDC1 and moDC subsets. *In vitro*, L-fucose treatment enhances antigen uptake and processing of DCs. Furthermore, our data suggests that L-fucose-treated DCs increase stimulation of T cell populations. Consistent with our functional assays, single-cell RNA sequencing of intratumoral DCs from melanoma- and breast tumor-bearing mice confirmed transcriptional regulation and antigen processing as pathways that are significantly altered by dietary L-fucose. Together, this study provides the first evidence of the ability of L-fucose to bolster DC functionality and provides rational to further investigate how L-fucose can be used to leverage DC function in order to enhance current immunotherapy.

## 1 Introduction

The myeloid compartment comprises a diverse subset of immune cells that can shape the immunological landscape in tumors (1–4). Broadly, myeloid cells (MCs) play key roles in mediating tumor suppression through early detection of tumor antigens, initializing recruitment of adaptive immune cells to the tumor microenvironment (TME) and elimination of pre-malignant and early-stage tumor cells (5, 6). As such, tumor-induced deregulation of the myeloid compartment can significantly influence tumor progression by stimulating tumorigenesis and enforcing an immunosuppressive microenvironment that undermines the efficacy of immunotherapies (7–10). Pro-tumorigenic MCs, such as myeloid-derived suppressor cells (MDSCs), regulatory dendritic cells (DC_reg_), tumor-associated macrophages and neutrophils (TAMs and TANs, respectively), represent an unique challenge for cancer immunotherapies. These populations promote tumorigenesis by abrogating anti-tumor immune function *via* suppressive mechanisms including attenuation or inhibition of intratumoral T cell activation and infiltration, promoting tumor antigen tolerance, and facilitating tumor metastasis (11–18). Thus, the immunosuppressive activities of these MCs can significantly contribute to poor clinical outcomes across a wide range of tumor types (19, 20). Recent studies have reported promising approaches for mitigating the immunosuppressive activities of these pro-tumorigenic MCs such as cytokine therapy (21, 22), immune depletion *via* gemcitabine (23) or reprogramming of populations through cytokines such as IFN-α/β and TNF-α (24, 25). Although these approaches have demonstrated the conceptual feasibility of therapeutically blocking immunosuppressive MC activities in tumors, they can result in a wide variety of adverse effects, including neuropsychiatric disorders (26), autoimmune disease onset (27), and off-target and deleterious co-morbidities observed in other bodily systems (28). For these reasons, novel therapeutic approaches to inhibit the development or activity of immunosuppressive MCs while sparing or promoting immunostimulatory MCs are urgently needed.

Dendritic cells (DCs) are a MC subset that is well established for its roles in regulating adaptive immune responses (29, 30). This MC subset is further subdivided by distinguishable phenotypic and/or functional characteristics. Conventional DC type 1 and 2 (cDC1 and cDC2) prime CD8^+^ and CD4^+^ T cell subsets, respectively, toward viral infections and cancer *via* antigen-loaded major histocompatibility complexes (MHC) to activate T cell responses towards the corresponding antigen of interest (30, 31). Monocyte-derived DCs (moDCs) are another major subtype of DCs, which can prime both CD8^+^ and CD4^+^ T cells, while also playing a key role in the innate inflammatory response (32). Dendritic cells are crucial for establishing effective and durable anti-tumor immune responses (33–36). A number of emerging anti-tumor immunotherapies leverage DC functions, such as DC vaccines (DCV), which comprises the pre-loading of DCs from a patient’s own DC repertoire with exogenous tumor antigens *ex vivo* (34, 37–40). This therapeutic modality allows for the priming of an abundance of DCs, which upon reinfusion, mediates a potent antitumor immune response that is mounted against tumor cells that present specific target antigens (38). However, DCV efficacy is often limited by tumor cells and pro-tumorigenic myeloid cells, which can abrogate anti-tumor DC function and maturation, causing release of tolerogenic cytokines, downregulation of antigen-presenting machinery, and upregulation of immunosuppressive molecules (41–44). Specifically, suboptimal *ex vivo* expansion and antigen uptake capacity of each individual patient’s DCs are major clinical challenges that limit DCV efficacy (10, 45). Thus, new therapeutic strategies that increase the proliferation and/or maturation of DCs *ex vivo* while maintaining or enhancing their functionality are urgently needed and expected to enhance clinical outcomes of DC-mediated therapies for patients.

L-fucose (L-fuc) is a dietary deoxyhexose plant sugar that is found in particularly high abundance in red and brown seaweeds (46, 47). In a process known as fucosylation, exogenous L-fuc is uptaken and processed in cells *via* the fucose salvage pathway and converted into GDP-L-fuc, which is conjugated onto proteoglycans in the ER or Golgi by 13 fucosyltransferases (FUTs) (48–52). In addition, GDP-L-fuc can also be cross-converted from intracellular GDP-mannose *via* the *de novo* synthesis pathway (53). Fucosylation can play crucial roles in regulating a range of processes including but not limited to cell:cell interactions, cell signaling, adhesion, protein folding, and unfolded protein response in normal and pathological biology (54, 55).

In cancer, increasing studies have reported associations of altered protein fucosylation or L-fuc levels with tumor presence or tumor progression. However, our mechanistic understanding of the functional and mechanistic contributions of altered levels of L-fuc or specific fucosylated proteins—as well as those of the individual FUTs—in tumorigenesis remains limited. Recent studies have however determined roles for a number of FUTs in the progression of cancer, which are currently under investigation as novel biomarkers or therapeutic targets. Briefly, reported examples include how FUT8 can mediate tumorigenesis by the increasing motility and potentially immune evasive capacity of melanoma cells (56), how FUT2 and 7 can increase proliferative capacity of lung cancer (57), and how FUT3 can exhibit a similar function in colorectal cancer (58). Interestingly, our group has recently shown that androgen can trigger upregulation of *Fut4*, inducing increased metastasis in men (59). While these studies illustrate the functional contributions of L-fuc and FUTs in tumorigenesis, we still lack a clear understanding of their precise mechanistic roles in the biology and functions of immune cell within the tumor microenvironment.

Given the emerging functional contributions of L-fuc to the biology of tumor cells, how protein fucosylation regulates the signaling and biology of immune cells—crucial microenvironmental regulators of tumor development, progression, and spread—while poorly understood, are the subject of increasing study. To present, few studies have reported the effect that L-fuc has on immune cells. However, fucosylation has previously been shown to be crucial for immune cell development and interactions. Several key immunomodulatory molecules — such as Notch1, IgG and the T cell receptor — are known to require fucosylation for proper function (60–63). These and other studies support the concept of therapeutic leveraging of fucosylation in immune cells to treat cancer (64–66). Our laboratory, which previously reported a role for L-fuc in restraining the metastatic capacity of melanoma cells (67), recently uncovered mechanistic roles for L-fuc in driving anti-melanoma immune responses. Specifically, we discovered that fucosylation of tumor-expressed MHC-II protein HLA-DRB1 triggers CD4^+^T cell-mediated induction of tumor-infiltrating immune cells and tumor suppression (68). Further tumor immunological profiling in this study revealed that L-fuc significantly alters DC subpopulations within the tumor and draining lymph node—an intriguing observation that prompted the initiation of this study. Although the precise mechanistic impact of L-fuc on DC biology and anti-tumor functions is unknown, our findings and others support the existence of a fucosylation-regulated mechanism that can modulate DC biology and promote their tumor-suppressive activities (69–71). Elucidation of such a pathway(s) is expected to identify novel therapeutic targets/modalities and provide key insight into signaling mechanisms in DCs that may have previously been understudied in the context of both immunology and immuno-oncology.

The use of L-fuc as a novel therapeutic agent has previously been reported by other groups to be a safe and effective option for patients—albeit not in cancer. Specifically, L-fuc has been studied as an experimental therapeutic agent for the treatment of leukocyte adhesion deficiency ll (LAD ll) (72). In this study, the authors observed no apparent adverse side effects to the oral administration of L-fucose at dosages of up to 492mg/kg. The use of up to ~1,400mg/kg/day of L-fuc for LAD ll is currently being investigated in 2 phase 3 clinical trials (#NCT05462587 and #NCT05754450). Additionally, the oral administration of L-fuc is being tested as potentially novel therapies for Alzheimer’s disease, to stimulate neurons under neurodeficient conditions (https://www.biorxiv.org/content/10.1101/2022.08.11.503673v1.full). Finally, previous studies have demonstrated that fucoidan, a plant-derived sulfated polymer of L-fucose, elicits apoptosis and prevents invasion in neck squamous cell carcinoma (73, 74). Additionally, a recent study has found that treatment with fucoidan can reverse the immunosuppressive re-education of macrophages in oral squamous cell carcinoma to stimulate an anti-tumor immune response (75). Thus, dietary fucoidan represents a potential alternate source of L-fuc-derived treatment. Together, these studies demonstrate that L-fuc is an effective and safe therapeutic agent for a variety of pathologies.

Here, we report that treatment with L-fuc enhances immunostimulatory activity, and notably, induces differential polarization of DCs, promoting myeloid maturation toward monocyte-derived DCs (moDC). This enhancement of immunostimulatory activity is characterized by the ability of DCs to increase T cell proliferation and activation. The L-fuc-treated DCs also exhibit increased antigen processing capacity and upregulated cell surface levels of MHC class ll. Single-cell RNA sequencing (scRNAseq) revealed that L-fuc upregulated genes associated with antigen processing and peptide loading, suggesting prominent upregulation of the antigen presentation pathway driving these observations. These data provide a preclinical rationale for the use of L-fuc to enhance DC functionality and its application to enhance DC-based therapeutic modalities, such as DCV. Additionally, our study is the first to our knowledge to investigate the global transcriptomic effects of L-fuc on intratumoral myeloid subsets, providing a breadth of novel mechanistic insight that future investigations may use to inform and enhance the efficacy of immunotherapies.

## 2 Materials and methods

### 2.1 Dendritic cell preparation

Dendritic cells were enriched from bone marrow (BM)-derived myeloid progenitors of BALB/c mice as previously described (76). Bone marrow cells were seeded at 1x10^6^ cells/mL and cultured in RPMI supplemented with 20ng/mL IL-4 [Peprotech (Cranbury, NJ)], 20ng/mL GM-CSF [Peprotech (Cranbury, NJ)], 10% fetal bovine serum [Sigma (St. Louis, MO)], and 10x Penicillin-Streptomycin (VWR) in the presence or absence of 250mM L-fuc [Carbsynth (San Diego, CA)] at 37°C in 5% CO_2_ unless otherwise noted. After 3 days, cells were supplied with fresh media with serum and additives. After 3 additional days, the cells were again supplied with fresh media and additives, including 10ug/mL LPS [Sigma (St. Louis, MO)]. After 24 hours, the treated cells were harvested for studies as indicated.

### 2.2 Mouse studies

Flow cytometric profiling of intratumoral immune subsets was performed on the following mouse model of breast cancer: 4-6-week-old female BALB/c mice were injected with 1x10^5^ 4T1 cells into the inguinal mammary fat pad. After 10 days, the mice were supplied with either control (n=5) or 500mM L-fuc (n=5)-supplemented water *ad libitum.* A treatment dosage of 500mM was chosen based on consistent maximal tumor suppression found in a L-fuc dose titration mouse model (**Supplementary Figure 2**) Tumor volumes were measured until the largest tumor reached endpoint (maximal volume of 1.5cm^3^), at which point all of the mice were sacrificed, and the tumors were subjected to flow cytometric profiling.

### 2.3 Single-cell RNA sequencing

For single-cell RNA sequencing of intratumoral immune cells, 4-6-weeks-old C3H/HeJ or BALB/c mice were injected subcutaneously into the right flank with 1x10^6^ SW1 melanoma cells or with 1x10^5^ 1T4 cells as above, respectively. After 14 days, a pretreatment mouse cohort (n=3) was sacrificed, and the harvested tumors were subjected to scRNAseq. The remaining mice were administered either control (n=6) or L-fuc (n=6; 100 or 500mM for SW1 or 4T1 models, respectively)-supplemented water *ad libitum* as previously described (67, 68). Tumors were harvested at 7 and 21 days after initiation of L-fuc treatment and subjected to scRNAseq analyses by 10X Genomics Chromium Single Cell Controller for single-cell RNA-sequencing library preparation [10X Genomics (Pleasanton, CA)]. Reverse transcription was then performed on encapsulated individual cell droplets to generate cDNA and generate cDNA libraries. These libraries were sequenced using an Illumina NovaSeq 6000 instrument with v1.5 Reagent kit for 100 cycles; a 10X Genomics CellRanger software was used for alignment and gene counting.

### 2.4 Single-cell RNA-sequencing data processing, clustering, and annotation

Sequencing reads were mapped against mm10 mouse transcriptome and processed for UMI counting using Cell Ranger (v3.0, 10X Genomics). Barcodes with UMI counts were imported to Seurat v4.0 (77) for downstream analysis. Cells with less than 200 genes detected, with greater than 10% mitochondrial UMIs, or with complexity score (log10GenesPerUMI) less than 0.8 were filtered out; genes detected in less than three cells were also excluded. Doublets were detected using Scrublet (78), DoubletFinder (79), and scDblFinder (80), using 0.08% doublet rate for every 1,000 cells. Cells identified as doublets by more than one method were removed. Raw UMI counts were log normalized and the top 5,000 variable genes were detected in each sample separately. S and G2/M cell cycle phase scores were assigned to cells using *CellCycleScoring* function. Individual mice samples were further integrated to remove batch effects using *IntegrateData* function (81) in Seurat with anchor.features =8,000. Scaled z-scores for each gene were calculated using *ScaleData* regressing against total reads count, mitochondrial UMIs percentage, cell cycle phases, and log10GenesPerUMI. Principal component analysis was performed on the integrated data and a shared nearest neighbor (SNN) graph was constructed using the first 40 principle components. Clusters were identified using the by Louvain clustering (82) implemented in *FindClusters* function at resolution=0.8. Uniform manifold approximation and projection (UMAP) was used to visualize gene expression and clusters. Differential expression analysis for each cluster was performed using *FindAllMarkers* function in Seurat with default settings. Clusters were further annotated by comparing differential genes with markers previously associated with T cells (*Cd3e, Cd3d, Cd3g*), B cells (*Cd79a, Cd79b, Cd19*), NK cells (*Gzma, Klrb1c, Ncr1*), cDC1 (*Xcr1*, *Clec9a*, *Itgae*, *Batf3*), cDC2 (*Lilrb4a*, *Itgax*, *Csf1r*, *Mgl2*), mregDC (*Fscn1*, *Ccl22*, *Cacnb3*, *Ccr7*, *Fabp3*), monoDC (*Cd7, Atp1b1, Clec10a, Cd209d*), Macrophages (*C1qc, C1qb, Apoe*), Monocytes (*Plac8, Ly6c2, Arg1*), Neutrophils (*S100a9, S100a8, Mmp9*), and Mast cells (*Cpa3, Hdc, Ifitm1*) (**Supplementary Figure 6C**). For marker gene bubble plot, gene-level average expression was calculated for each cluster and then Z-score normalized.

### 2.5 Differential expression and gene set enrichment analysis

To systematically identify effects of L-fucose, differential expression analysis was performed followed by gene set enrichment analysis (GSEA) that compared cDC1 populations within L-fuc-fed vs. control mice. Volcano plot was used to show differential expression results (**Supplementary Figure 6D**). Genes were ranked based on -log10(p-value)*(sign of log2(fold-change)) resulted from the differential analysis, with most up-regulated genes at the top and most down-regulated genes at the bottom. Pre-ranked GSEA was performed on gene rankings using R package fgsea (83) with 10,000 permutations, against REACTOME databases from MsigDB (84–86).

### 2.6 Antibodies

The following antibodies were used for experiments involving flow cytometric analyses as indicated in the main text and Figure Legends: FITC rat anti-F4/80 [0.4ug/uL, Biolegend (San Diego, CA)], BV711 rat anti-I-A/I-E [0.4ug/uL, BD Biosciences (San Jose, CA), PE rat anti-perforin [0.4ug/uL, Biolegend (San Diego, CA)], PE anti-CD209 [0.4ug/uL, Tonbo Biosciences (San Diego, CA)], PE/Dazzle mouse anti-FCγlll [0.4ug/uL, Biolegend (San Diego, CA)], APC rat anti-Ly6G [0.4ug/uL, Biolegend (San Diego, CA)], BV605 rat anti-ly6C [0.4ug/uL, Biolegend (San Diego, CA)], BV510 mouse anti-XCR1 [0.4ug/uL, Biolegend (San Diego, CA)], PerCP/Cyanine5.5 rat anti-CD40 [0.4ug/uL, Biolegend (San Diego, CA)], BV650 hamster anti-CD103 [0.4ug/uL, BD Biosciences (San Jose, CA)], PE/Cy7 American Hamster anti-CD11c [0.4ug/uL, Biolegend (San Diego, CA)], FITC rat anti-CD45 [0.4ug/uL, BD Biosciences (San Jose, CA)], BV421 rat anti-CD8a [0.4ug/uL, BD Biosciences (San Jose, CA)], BV605 rat anti-CD86 [0.4ug/uL, BD Biosciences (San Jose, CA)], APC rat anti-GR-1 [0.4ug/uL, Biolegend (San Diego, CA)], BV785 rat anti-CD8a [0.4ug/uL, Biolegend (San Diego, CA)], BV421 rat anti-CD103 [0.4ug/uL, BD Biosciences (San Jose, CA)], APC-R700, rat anti-F4/80 [0.4ug/uL, BD Biosciences (San Jose, CA)], BV785 rat anti-CD11b [0.4ug/uL, Biolegend (San Diego, CA)], PE rat anti-H2 [0.4ug/uL, Biolegend (San Diego, CA)], and APC rat anti-CD45 [0.4ug/uL, Biolegend (San Diego, CA)].

### 2.7 In vitro studies

Unless otherwise indicated, BM-derived DCs were derived as indicated above for the following *in vitro* assays:

### 2.8 Neutral bead uptake

Control- and L-fuc-treated DCs were incubated with 10mg/mL of Fluorescent Polystyrene Microspheres [Lab 261 (Palo Alto, CA)] for 8 hours at 4 or 37°C in RPMI. Cells were then washed 3 times with PBS, fixed in 5% formaldehyde, and analyzed by flow cytometry.

### 2.9 Dendritic cell antigen processing

Control- and L-fuc-treated bmDCs were incubated with 5ug/mL of DQ-OVA (Invitrogen (Waltham, MA) for 30 mins at 37°C in RPMI as previously described (87). The DQ-OVA-containing media was then removed and cells were then washed 3 times with PBS and incubated for the indicated time at 37°C in RPMI. At the indicated timepoints the cells were then fixed with 5% PFA and analyzed by immunofluorescence.

### 2.10 Dendritic cell antigen uptake

Control- and L-fuc-treated bmDCs were incubated concurrently with 5ug/mL of DQ-OVA [Invitrogen (Waltham, MA)] and 50nM LysoTracker^®^ [ThermoFisher Scientific (Waltham, MA)] for 30 mins at 37°C in RPMI as previously described (87). The DQ-OVA- and LysoTracker^®^-containing media was then removed, and cells were then washed 3 times with PBS before being fixed with 5% PFA and analyzed by immunofluorescence.

### 2.11 Dendrite length

Dendritic cells were cultured on glass coverslips coated with 10ug/mL of fibronectin [Sigma-aldrich (St. Louis, MO)]. After 6 days of culture, cells were stained with Phallodin 488 [1:300 dilution; Caymen Chemical company (Ann Arbor, MI)]. Images of the cells were acquired using a Keyence BZ-X700 fluorescent microscope [Keyence (Itasca, IL)], and the resulting images were processed, and dendrite lengths (as defined by length of protrustion from the soma) were measured using FIJI (NIH, Version: 2.3.0/1.53q).

### 2.12 Analysis of cytokine production

Supernatants from cultured DCs were harvested and analyzed for secreted cytokines as indicated below unless otherwise noted. For IFNγ release, supernatants from T cells cultured in the presence or absence of treated DCs were analyzed using ELISA MAX kits [Biolegend (San Diego, CA)]. To measure nitrite production, supernatants were subjected to the Griess Test assay [Invitrogen (Waltham, MA)]. To study cytokine production, supernatants were analyzed using the LEGENDplex system [Biolegend (San Diego, CA)], followed by flow cytometric analysis using Biolegend software.

### 2.13 Flow cytometry

Cells were harvested and washed with PBS on ice. Cells were then blocked in 0.5% BSA for 30 minutes on ice, followed by labeling with indicated antibodies for 30 minutes on ice, and lastly, the cells were fixed in 2% PFA for 30 minutes on ice. Expression of indicated markers were detected using a BD LSR ll flow cytometer [BD Biosciences (San Jose, CA)] followed by FlowJo software analysis. Gating strategies are outlined in **Supplementary Figure 1**.

### 2.14 qRT-PCR

RNA was extracted from cells treated as indicated using the Gene Elute Mammalian Total RNA Extraction System [Millipore Sigma (St. Louis, MO)]. cDNA was generated from the RNA using the High-Capacity cDNA Reverse Transcription Kit [ThermoFisher Scientific (Waltham, MA)]. qRT-PCR analysis was then performed using the FastStart Universal SYBR Green Master Mix [Roche Diagnostics, (Indianapolis, IN)] and a BioRad CFX96 Real-time system [BioRad Laboratories, (Hercules, CA)]. Unless otherwise indicated, the following cycle was used for qRT-PCR: 95°C for 10 min and 35 cycles of 95°C for 15 seconds, 55°C for 60 seconds, and 72°C for 30 seconds. Gene expression was normalized to *H3A* histone levels of the indicated control samples. The primers were generated using the NCBI primer blast software [IDT (Coralville, Iowa)] (**Supplementary Table 1**).

### 2.15 Quantification and statistical analysis

GraphPad Prism was used for statistical analyses. Comparisons between 2 treatment groups were made using Student's T-test and reported as p-values and standard errors of the mean (SEM). Unless otherwise noted, all experiments were performed at minimum in independent biological triplicate.

## 3 Results

### 3.1 L-fucose increases the abundance and alters the polarization of intratumoral dendritic cells

**Figure 1:**
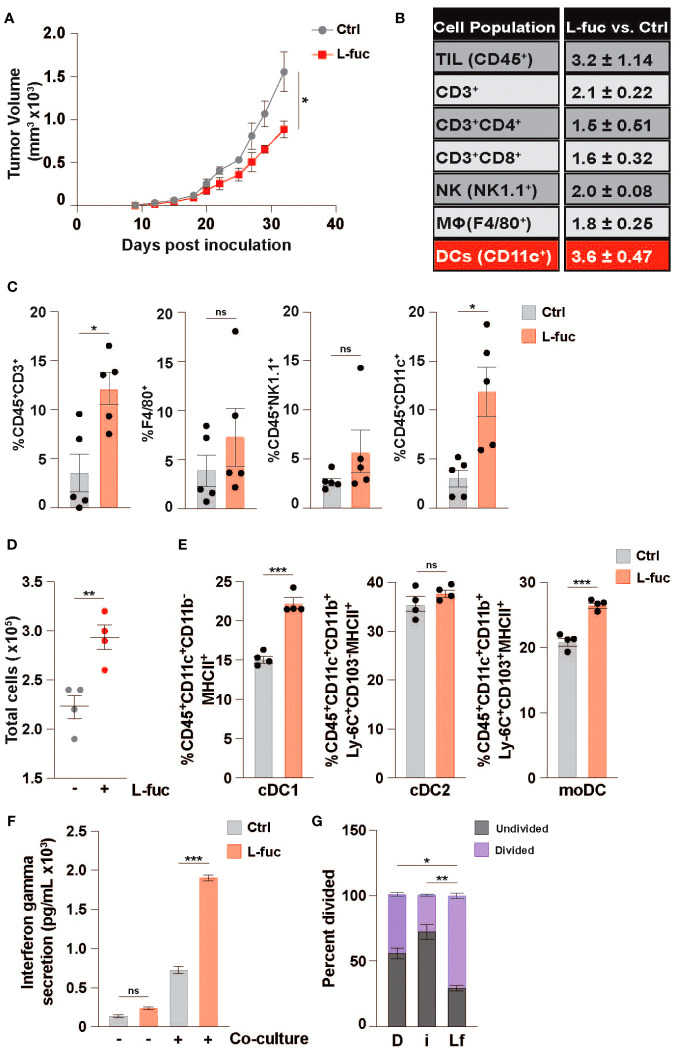
L-fucose increases the abundance of specific dendritic cell subsets. **(A)** Growth curves of 4T1 tumors in mice fed ± 500mM L-fuc. (n = 5 mice per cohort, *= p < 0.05, error presented as SEM). Flow cytometric profiling of tumors from **(A)** for the indicated immune populations showing **(B)** fold-changes and **(C)** % indicated immune cell population of total CD45+ lymphocytes in L-fuc vs. Ctrl groups (n = 5 per cohort, * = p < 0.05, ns = not significant, error presented as SEM). **(D)** Total bone-marrow-derived DC counts (representative figure of 3 replicates, n = 4, ** = p < 0.01, error presented as SEM). **(E)** Flow cytometric analyses of cDC1 (CD45+CD11c+CD11b-MHC ll+), cDC2 (CD45+CD11c+CD11b+Ly-6C+CD103-MHC ll+), or moDC (CD45+F4/80-CD11c+CD11b+Ly-6C+CD103+MHC ll+) subtype abundance resulting from Ctrl- or L-fuc-treated bmMCs after 6 days (representative figure of 3 replicates, n = 4, *** = p < 0.001, ns = not significant, error presented as SEM). **(F)** ELISA showing increased Interferon-γ secretion of T cells after 48 hours of co-culture with DCs that were cultured ± antigen loaded L-fuc after 48 hours (representative figure of 3 replicates, n = 3, *** = p < 0.001, ns = not significant, error presented as SEM). **(G)** Flow cytometric assessment showing altered cell trace violet staining of T cells after 72 hours of co-culture with DCs treated prior to co-culture with DMSO **(D)**, fucosyltransferase inhibitor 2FF (i) or L-fuc (Lf) for 6 days (representative figure of 3 replicates, n = 3, * = p < 0.05, ** = p < 0.01, error presented as SEM).

Based on our recent study demonstrating that L-fuc can promote a tumor-suppressive immune landscape in melanomas (68), we sought to determine if the apparent anti-tumor immunological effects are conserved in other tumor types, particularly those that are generally considered as immunosuppressive tumor microenvironments (i.e., breast cancer). To explore this possibility, we implanted female BALB/c mice with 1x10^4^ syngeneic 4T1 triple negative mouse breast tumor cells and fed the mice ± 500mM L-fuc, a concentration that we found to induce similar levels of suppression of the 4T1 tumors as in our previous melanoma models (**Figure 1A**; **Supplementary Figure 2**). Importantly, we found that L-fuc also induced the accumulation of intratumoral immune cells, including CD3^+^ T cells (**Figures 1B, C**). Intriguingly, in this breast tumor model, although T cells were significantly increased, the intratumoral immune cell subset that was most increased by L-fuc was CD11c^+^ DCs (**Figures 1B, C**). As oral L-fuc is a systemic treatment that may elicit a range of direct and indirect effects on multiple other cell types, it is important to establish the effect of L-fuc on individual cell populations to gain a better understanding of how to properly leverage L-fuc treatment in the future by removing factors that may affect DC function in the tumor microenvironment or by other immune cells. To this end, we first assessed the potential effects of L-fuc on DCs derived from bone marrow (BM) myeloid progenitor cells of healthy BALB/c mice as this would provide us with information on how L-fuc interreacts with DCs to establish a precedent that future studies can apply to harnessing the function of L-fuc on intratumor DCs. At 7 days of treatment after initial BM isolation, BM myeloid progenitors treated concurrently with IL-4 and GM-CSF to stimulate DC maturation (76) and L-fuc produced significantly more DCs compared with progenitors stimulated with IL-4 and GM-CSF alone (**Figure 1D**), suggesting that L-fuc enhances DC proliferation. Flow cytometric profiling revealed that L-fuc treatment increased the abundance of each major DC subset (DC1, DC2, and monocyte-derived DC (moDC)), although interestingly, the cDC1 and moDC subsets were most increased (**Figure 1E**). Additionally, we found that the culture conditions used to generate bmDCs yielded minimal numbers of macrophages that were unaffected by L-fuc (**Supplementary Figure 3**), indicating that our use of bone marrow-derived DCs (bmDCs) is an effective method to define DCs in our studies. Together, these findings revealed that L-fuc modulates DC abundance *in vitro* and *in vivo* and alters subtype differentiation of DCs. Given the significant increases in abundance and subtype differentiation observed after L-fuc treatment, we sought to determine if L-fuc alters the ability of the DCs to process antigen and activate T cells—key functions that might help to mechanistically explain contributions of DCs in L-fuc-mediated enhanced functionality. To functionally determine if L-fuc, and more broadly, fucosylation significantly alters the ability of DCs to activate T cells, we co-cultured control or fucosylation-modulated bmDCs with peripheral T cells and measured T cell interferon gamma (IFNγ) release and proliferation. To this end, we cultured DCs in media supplemented with a mimic of L-fuc, 2FF, which inhibits FUTs, abrogating cellular fucosylation of proteins (88) or with L-fuc (to increase fucosylation) as described. Treatment of bmDCs with L-fuc prior to co-culture with T cells augmented T cell activation, as reflected by increased IFNγ release and T cell proliferation (**Figures 1F, G**, respectively). In contrast, treatment with 2FF reduced T cell proliferation. Together, our findings suggest that L-fuc may enhance DC-mediated T cell activation by increasing fucosylation of key proteins that regulate DC biology. Future studies are warranted to investigate the specific proteins that are fucosylated in DCs to further verify this possibility. These data suggest that pre-treatment of the bmDCs with L-fuc increased T cell proliferation and IFNγ secretion, suggesting that L-fuc augments the immunostimulatory functions of DCs—including antigen uptake and presentation.

### 3.2 L-fucose increases antigen processing efficiency in dendritic cells

**Figure 2:**
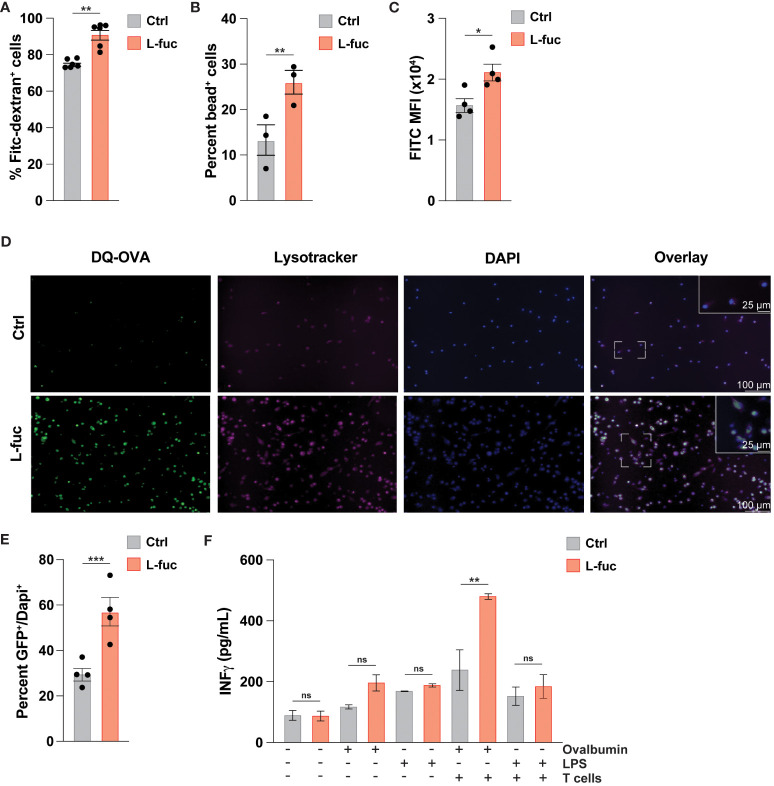
L-fucose increases antigen uptake capacity of dendritic cells. **(A-C)** Flow cytometric analysis of FITC signal from **(A)** FITC-Dextran, **(B)** fluorescent nanoparticles or **(C)** cleaved DQ-OVA in bmDCs treated ± L-fuc for 6 days and incubated with DQ-OVA for 1 hour to permit early antigen uptake (representative figure of 4 replicates, n = 3, * = p < 0.05, ** = p < 0.01, error presented as SEM). **(D)** Representative immunofluorescent microscopy (20x and 40x) images showing bmDCs that were treated ± L-fuc for 6 days and incubated with DQ-OVA and Lysotracker™ for 1 hour. **(E)** The number of FITC+ foci per total number of DAPI+ cells in **(D)** were assessed using Fiji (representative figure of 4 replicates, n = 4, *** = p < 0.001, error presented as SEM). **(F)** ELISA showing interferon-γ secretion from OT-ll T cells that were co-cultured with bmDCs treated ± L-fuc for 6 days and loaded with the indicated antigen for 48 hours (representative figure of 3 replicates, n = 3, ** = p < 0.01, ns = not significant, error presented as SEM).

As DC-mediated T cell activation is facilitated by the processing and presentation of antigens we next sought to assess how L-fuc might affect antigen uptake by DCs. To this end, we cultured bmDCs treated ± L-fuc with either fluorescently tagged dextran or neutral polyurethane nanobeads and observed increased uptake of both fluorescent baits in the L-fuc-treated bmDCs (**Figures 2A, B**). To ensure that the observed increases in fluorescent bait uptake are reflective of more physiological antigen uptake, we cultured control- or L-fuc-treated bmDCs in the presence of DQ-OVA, a fluorescently-quenched model antigen that can be used to assess antigen phagocytosis and processing. After uptake into cells, the fluorescence-quenching subunit attached to the OVA molecule is cleaved during proteasomal degradation in the lysosome, permitting fluorescence that is detectable and quantifiable by microscopy or flow cytometry as an indicator of antigen processing activity in DCs (89). Flow cytometric analyses revealed an increase in the percentage of fluorescent OVA^+^ cells upon treatment with L-fuc (**Figure 2C**), indicating that treatment with L-fuc increases the phagocytotic activity of DCs. To further confirm that L-fuc increases antigen processing activity in DCs, we assessed the subcellular localization of the fluorescent signal of DQ-OVA. As antigen processing occurs in the lysosome of DCs shortly after antigen uptake (90), we treated the DCs ± L-fuc concurrently with DQ-OVA and Lysotracker™, a lysosome marker (91), for 1 hour prior to imaging to visualize early antigen processing in the DCs. As we had previously observed, L-fuc significantly increased the number of DQ-OVA^+^ DCs. Further, that the FITC^+^ OVA and Lysotracker™ signals exhibited significantly more subcellular colocalization in L-fuc-treated DCs confirms that the cleavage of DQ-OVA occurred in the lysosomes of DCs during early antigen processing (**Figures 2D, E**). Surprisingly, while we observed no difference in the total number of MHC^+^ DCs, DCs treated with L-fuc exhibit an increased abundance of MHC molecules per cell (**Supplementary Figure 4**). These data suggest that L-fuc treatment increases the rate of exogenous antigen uptake and processing in DCs, consistent with increased abundance of antigen-presenting MHC complexes on the cell surface that would be expected to mediate the enhanced T cell priming/activation that we observed in **Figures 2A, B**. Accordingly, L-fuc treatment enhanced the ability of DQ-OVA-treated DCs to induce the IFNγ-producing activity of co-cultured OT-l T cells, confirming that L-fuc enhances cell surface antigen presentation by T cells (**Figure 2F**). Together, these data demonstrate that L-fuc enhances antigen processing/presentation and T cell priming by DCs—effects with significant translational implications for enhancing the efficacy of existing DC-based immunotherapies.

### 3.3 L-fucose alters the transcriptional profile of dendritic cells

**Figure 3:**
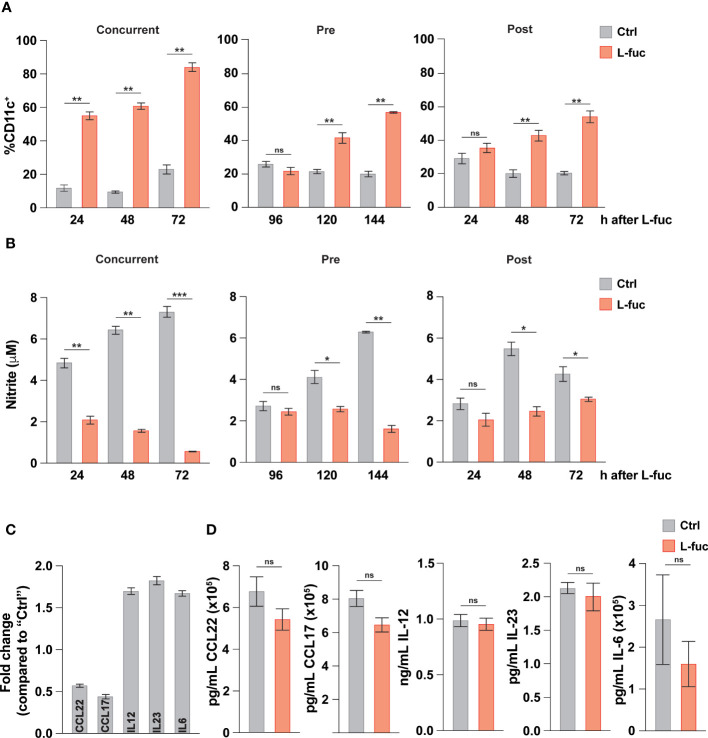
L-fucose transcriptionally alters dendritic cells to be immunostimulatory. **(A)** Flow cytometric analysis of total DC abundance after L-fuc treatment of bmDCs as indicated in (**Supplementary Figure 5**) (representative figure of 3 replicates, n = 3, * = p < 0.05, ** = p < 0.01, ns = not significant, error presented as SEM). **(B)** Griess assay of nitrite secretion by bmDCs treated as indicated in (**Supplementary Figure 5**) (representative figure of 3 replicates, n = 3, * = p < 0.05, ** = p < 0.01, *** = p < 0.001, ns = not significant, error presented as SEM). **(C)** qPCR of immunomodulatory genes in bmDCs treated + L-fuc for 6 days compared to Ctrl treated bmDCs (representative figure of 3 experiments, n = 3, error presented as SEM). **(D)** LEGENDPlex™ analysis of cytokine production in bmDCs treated ± L-fuc for 6 days followed by 24 hours of antigen treatment (representative figure of 4 replicates, n = 3, ns = not signficiant, error presented as SEM).

Intratumoral DCs mature from tumor-infiltrating myeloid cells in a continuum of maturation. Thus, the observed effects of L-fuc likely represent the collective responses of DCs in multiple stages of myeloid development that are impacted by L-fuc within the tumor microenvironment. Thus, we delineated the effects of L-fuc on myeloid cells at different stages of maturation by treating the cells with L-fuc over a time course of myeloid development. To this end, we treated immature BM-derived myeloid progenitors with L-fuc during multiple stages of IL-4 and GM-CSF-stimulated DC maturation as follows: (i) for 72 hours prior to, (ii) during, or (iii) for 72 hours after maturation induction (**Supplementary Figure 5**). Surprisingly, regardless of the timing of L-fuc treatment, we observed that L-fuc treatment increased the proportion of CD11c^+^ DCs from BM-derived myeloid progenitors (**Figure 3A**), suggesting that L-fuc affects the polarization of myeloid cells through a common lineage (92). Further, consistent with a L-fuc-induced shift toward immunostimulatory phenotype, L-fuc significantly reduced the capacity of DCs to produce nitrite, a molecule that is secreted by immunosuppressive myeloid cells (**Figure 3B**). To further validate our previous findings that L-fuc induces an immunostimulatory phenotype in DCs, we performed qRT-PCR on control- vs. L-fuc-treated myeloid cells for common DC immunostimulatory and immunosuppressive markers. Consistent with our functional findings, L-fuc treatment resulted in the reduced expression of markers that characterize immunosuppressive myeloid activity (*CCL22* and *CCL17*) and concomitantly increased expression in immunostimulatory myeloid markers (*IL-6, -12* and *-23)*, suggesting that L-fuc triggers a dynamic transcriptional shift towards an immunostimulatory phenotype, which promotes the accumulation and activation of T cell subsets over time (**Figure 3C**). Surprisingly, when we measured the secretion of these cytokines under the same conditions, we did not observe any changes in concentration of cytokines present in the supernatant as detected by Legendplex™ (**Figure 3D**). The lack of detectable cytokines could be attributed to a temporal disparity between when transcriptional profiles are altered by L-fuc and when cytokine production and release occurs in DCs. In line with our previous experiments, cytokine release was measured 24 hours after antigen treatment, however it is possible that observable changes in cytokine release occur beyond that point. Another potential reason is that given that the cells used in this study were generated *ex vivo*, the appropriate conditions for cytokine release were not met, resulting in suboptimal release of cytokines across the conditions (93). Additional studies are needed to analyze changes in cytokine production in L-fuc-treated DCs under immunologic conditions. Together with our findings above, these data suggest that although L-fuc promotes polarization of DCs toward immunostimulatory cDC1s and moDCs, the effects, while apparent at the transcriptional level, appear to have some limitations at the post-transcriptional level at least in terms of the secretion of these cytokines.

### 3.4 L-fucose modulates dendritic cell phenotypes in tumor-bearing conditions

**Figure 4:**
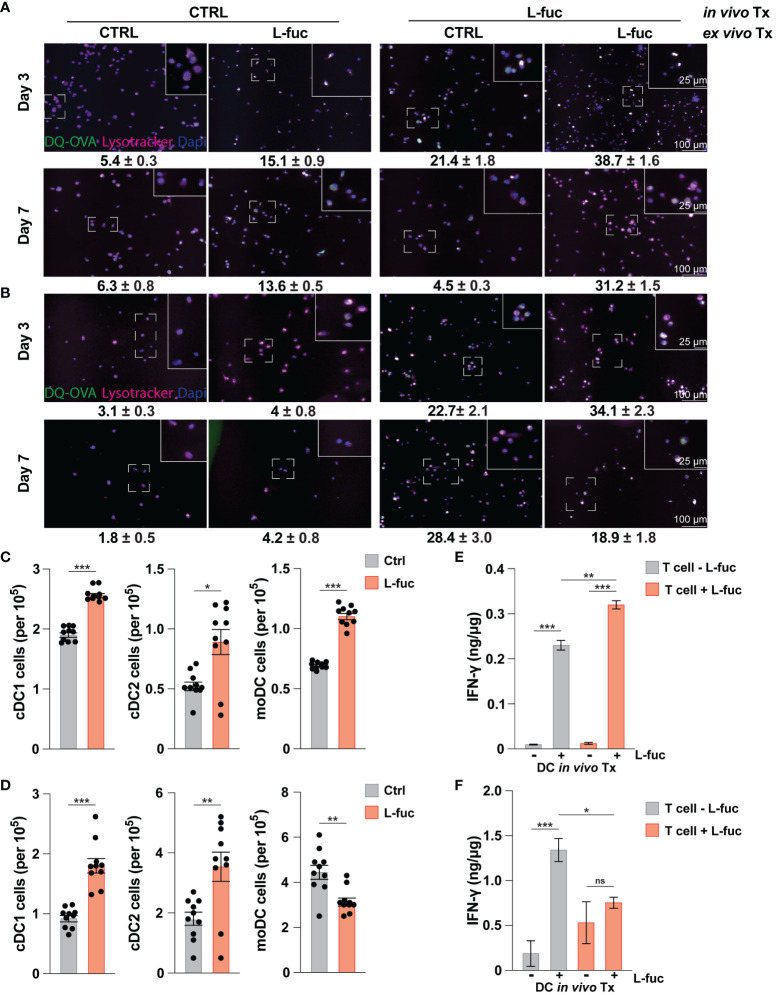
The increased immunostimulatory effect of L-fucose on dendritic cells is conserved in tumor bearing mice. **(A, B)** Representative immunofluorescent imaging of bmDCs harvested at either **(A)** 7 or **(B)** 21 days after initiation of *in vivo* L-fuc treatment and treated as indicated ex vivo for 6 days prior to incubation with DQ-OVA and Lysotracker™ for 1 hour with corresponding means of GFP+ cells/Dapi^+^ cells (representative images of 3 replicates, error presented as SEM). **(C, D)** Flow cytometric analysis of intratumoral DC subsets **(C)** 7 and **(D)** 21 days after initiation of L-fuc treatment (representative figure of 3 replicates, n = 5 mice per cohort, * = p < 0.05, ** = p < 0.01, *** = p < 0.001, error presented as SEM). **(E, F)** Interferon-γ ELISA of splenic-derived DCs harvested **(E)** 7 and **(F)** 21 days after initiation of L-fuc treatment and co-cultured as indicated for 48 hours (representative figure of 2 replicates, n = 5 per treatment condition, * = p < 0.05, ** = p < 0.01, *** = p < 0.001, ns = not significant, error presented as SEM).

Our data above demonstrates that L-fuc can promote an immunostimulatory phenotype in bmDCs, characterized by increased antigen uptake and processing capacity, enhanced ability to activate T cells, and polarization towards cDC1 and moDC subsets. As we have previously sought to understand how L-fuc and DCs interact without the added variables present in the TME, we next sought to understand if these effects were conserved among intratumoral DCs. To this end, 4T1 tumor cells were injected into the mammary fat pads of female BALB/c mice, and the resulting tumors, spleen and BM were harvested at 7 and 21 days after L-fuc treatment. As MCs are a heterogeneous group of cells that can accumulate within the TME at various points of maturation, we sought to understand how systemic treatment with L-fuc affects intratumoral, splenic, and bone marrow MCs in terms of their ability to process antigen and fully differentiate and mature into DCs. To elucidate the systemic effect of L-fuc in tumor-bearing mice on MCs, BM was harvested at the indicated timepoints from mice treated ± L-fuc and cultured for either 3 or 7 days before OVA uptake was measured as preformed in our experiments in **Figure 2**. Additionally, the cells were cultured ± L-fuc to establish if the observed enhanced immunostimulatory capacity is sustained after the MCs are harvested from the BM. Not surprisingly, bmDCs that were harvested from control-fed mice and treated with L-fuc *ex vivo* resulted in a significantly higher number of OVA^+^ cells compared to bmDCs that never experienced L-fuc treatment. Interestingly and in contrast, bmDCs harvested from L-fuc-fed mice that were treated further with L-fuc *ex vivo* did not exhibit as significant of an increase in the number of OVA^+^ cells compared to bmDCs that only experienced L-fuc *in vivo* from feeding (**Figures 4A, B**), indicating that the effect of L-fuc is persistent in bmDCs even after cessation of treatment. We next assessed the effect of short- versus long-term L-fuc on intratumoral DCs by analyzing intratumoral DC subtypes present at 7 and 21 days after L-fuc treatment. At 7 days after L-fuc treatment, we observed an increase in each of the 3 DC subtypes (**Figure 4C**). However, at 21 days after treatment, whereas both cDC1 and cDC2 subsets still exhibited increases in the tumors of L-fuc-fed, interestingly the intratumoral moDCs decreased in the L-fuc-fed mice (**Figure 4D**). Finally, to simulate tumor antigen uptake in the TME by immature DCs, splenic DCs were harvested at 7 and 21 days and cultured with lysed 4T1 tumor cells. The antigen-treated DCs were then co-cultured with T cells from corresponding mice treated ± L-fuc, and IFNγ release was measured by ELISA to determine if systemic L-fuc treatment affected T cell activation primarily through the DCs. To this end, we observed that regardless of the L-fuc treatment status of T cells, L-fuc treatment of DCs resulted in significant production of IFNγ release from both control- and L-fuc-treated T cells. Interestingly, at day 21, we did not observe an increase in IFNγ release when both the DCs and T cells were treated with L-fuc (**Figures 4E, F**). These data suggest that the treatment of splenic DCs with L-fuc promotes antigen uptake and presentation and leads to increased IFNγ release by T cells, regardless of L-fuc treatment. Taken together, our findings indicate that L-fuc treatment can alter the biology of MCs in a way that promotes the accumulation of intratumoral DCs and enhances early antigen uptake and processing in BM-derived and splenic DCs.

### 3.5 L-fucose promotes upregulation of antigen-processing associated genes

**Figure 5:**
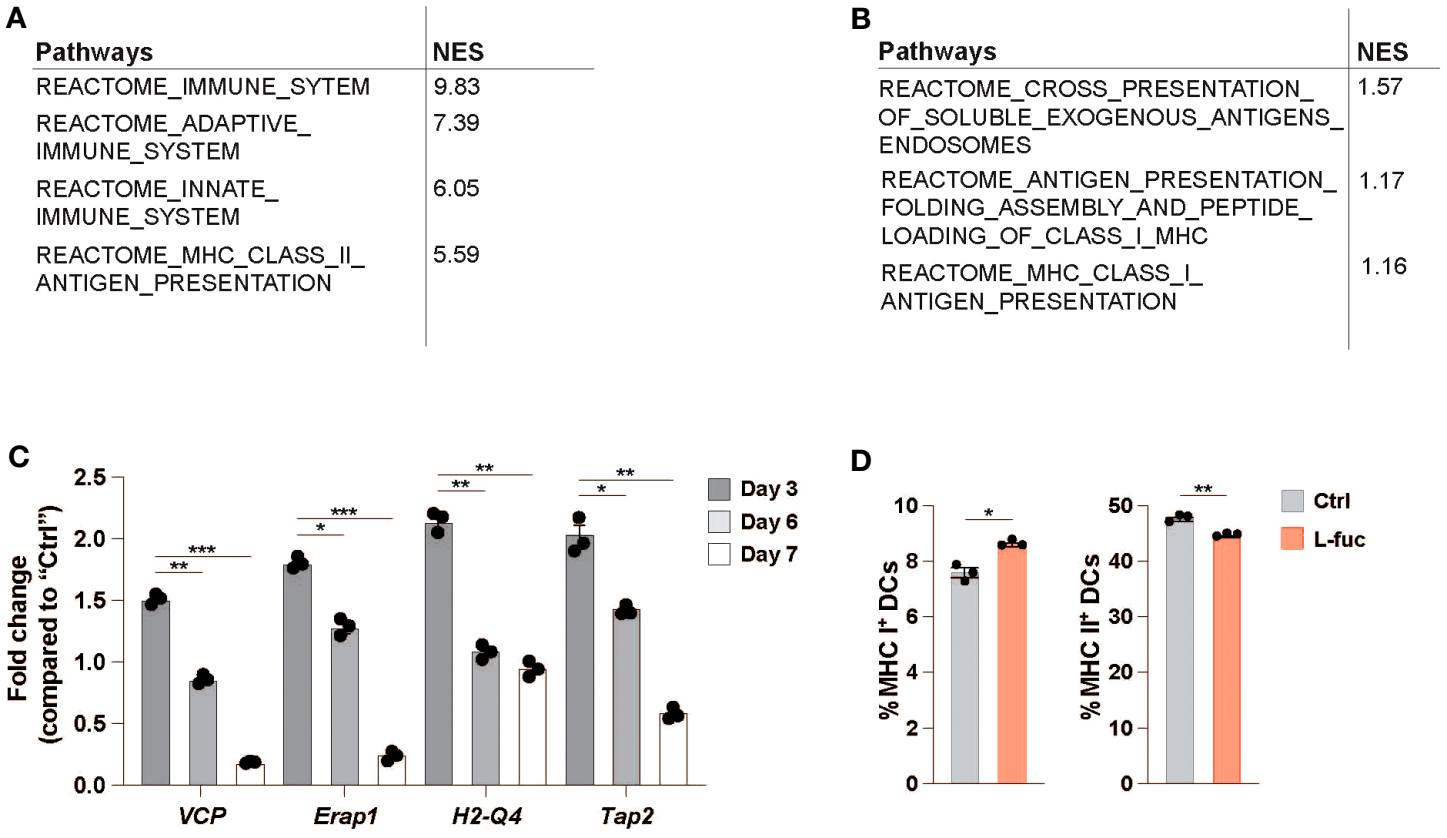
Single-cell RNA sequencing identifies antigen-processing is upregulated in L-fucose-treated dendritic cells. **(A, B)** Reactome pathway analysis of antigen processing-related pathways altered in L-fuc-treated DCs compared to control treated mice 7 days after L-fuc treatment in SW1 and 4T1 tumors respectively. **(C)** qRT-PCR validation of genes associated with antigen presentation in L-fuc-treated bmDCs compared to control-treated cells (representative figure of 3 replicates, n = 3, * = p < 0.05, ** = p < 0.01, *** = p < 0.001, error presented as SEM). **(D)** Flow cytometric analysis of cross presentation in bmDCs treated ± L-fuc for 6 days prior to 24 hours of antigen treatment (n = 4, * = p < 0.05, ** = p < 0.01, error presented as SEM).

In addition to flow cytometric analysis of dendritic cells in the TME, we performed single-cell RNA sequencing (scRNAseq) to understand the systemic effects that L-fuc treatment has on intratumoral DCs. To this end, SW1 melanoma and 4T1 breast tumors from female CH3/Hej and BALB/c mice, respectively, treated ± L-fuc were harvested 7 and 21 days after initiation of L-fuc treatment and analyzed by scRNAseq to establish changes in dendritic cells specific transcription. Clusters of CD45^+^ identified relevant immune populations isolated at each timepoint (**Supplementary Figures 6A, B**), including several DC subsets which were identified using previously established markers (**Supplementary Figure 6C**). Our group’s previous work (68), as well as our *ex vivo* data analyzing both bmDC (**Figure 1E**) and intratumoral DCs (**Figures 4C, D**) show that L-fuc-treatment increases the abundance of cDC1s, which is further confirmed by our initial scRNAseq screen at 7 days after L-fuc treatment (**Supplementary Figure 6B**). As these data strongly support the rationale that L-fuc affects the immunostimulatory potential of cDC1s, we sought to focus our scRNAseq analysis on this DC subset to further demonstrate the conserved nature of our findings between *ex vivo* and intratumoral DCs. Upon analysis of transcriptional changes in cDC1s from L-fuc-treated mice we observe increased transcription of genes in the antigen presentation and antigen cross presentation pathways in both tumor models, in line with our *ex vivo* findings (**Figures 5A, B**, **Supplementary Table 2** and **Supplementary Figure 6D**). Surprisingly, the changes in antigen presentation identified by scRNAseq in cDC1 cells suggested L-fuc-induced alterations in MHC-l-mediated antigen presentation as well as MHC-ll-mediated antigen presentation, specifically as indicated by changes in the expression of *VCP, Erap1, H2-Q4* and *Tap2*. Importantly, the expression of these genes, for which we validated L-fuc-induced changes in bmDCs (**Figure 5C**), has been reported to play roles in DC cross-presentation (94–98). Interestingly, the expression of the indicated genes decreases over time during bmDC maturation (**Figure 5C**), consistent with myeloid cell maturation to DCs (99), suggesting that L-fuc-treated bmDCs mature over a shorter time than control-treated cells, however further studies are needed to elucidate this phenomenon further. We evaluated whether cross-presentation occurs in the L-fuc-treated DCs by flow cytometry. We performed flow cytometry to assess levels of both MHC class I and class II, both of which are needed in order for DCs to effectively cross-present antigen (100, 101). Intriguingly, L-fuc-induced changes in expression levels of both MHC l and ll in bmDCs were surprisingly minor (**Figure 5D**) compared to our earlier findings (**Figure 1E**). This discrepancy is likely attributed to the latter assessment of a pool of all DCs that contains a predominance of cDC2s as opposed to the earlier flow-sorted analysis of individual DC subsets. Nonetheless, the L-fuc-increased *in vivo* anti-tumor immunity (**Figure 1A**) and the T cell activation levels (**Figures 2**, **4E, F**) are certainly attributed to additional L-fuc-regulated factors beyond MHC complex expression/presence that are associated with cross-presentation, such as antigen uptake and processing, which we have shown to be increased by L-fuc (**Figures 2A–E**). Future studies are warranted to investigate if and how L-fuc may alter antigen binding in both MHC classes and facilitates cross-presentation in DCs. Together our data indicate that treatment with L-fuc, which is associated with marked transcriptional changes associated with antigen presentation, increases antigen uptake and processing in DCs that likely mediate L-fuc-triggered immune responses and could be leveraged to initiate tumor suppression.

## 4 Discussion

The use of L-fuc as a modulator of anti-tumor immunity is a novel concept which was previously shown to depend on the fucosylation of a tumor-expressed MHC class ll molecule and CD4^+^ T cells (68). In this study, we expand on these findings by demonstrating a novel mechanism by which L-fuc causes the polarization and accumulation of DCs that can better facilitate an immunostimulatory response through enhance antigen uptake, processing and presentation. These findings represent a novel and potentially safe way to leverage potent and immunostimulatory DCs to robustly increase the efficacy of current immunotherapies, particularly dendritic cell vaccines (DCVs). Taken together, our findings that L-fuc leverages anti-tumor immunity through both CD4^+^ T cells (68), and now potentially DCs, suggest that the treatment of cancer with oral L-fuc can bolster a patient’s anti-tumor immune responses and provide preclinical rationale to combine L-fuc with DC-mediated immunotherapeutic approaches. However, future studies are needed to elucidate the relative effects of L-fuc on DCs in tumor microenvironment.

Although our findings demonstrate that L-fuc increases immunostimulatory potential in DCs, what DC proteins interact with L-fuc, or are themselves fucosylated, to alter DC signaling and biology is unknown. Our scRNAseq data revealed that C-type lectin receptor (CTLR)-related signaling pathways are upregulated after L-fuc treatment. Notably, several CTRL family members are capable of binding to fucosylated structures (102). Specifically, the CTLR dendritic cell-specific intracellular adhesion molecule-3-grabbing non-integrin (DC-SIGN/CD209) is highly expressed on both macrophages and monocyte-derived dendritic cells (moDCs) and binds CD15, a fucosylated glycan (103). While CD209 can interact with fucosylated proteins, less is known about whether it can interact with free L-fuc in the extracellular environment. Interestingly though CD209 is predominantly expressed on moDCs, one of the DC subpopulations that we found to be most significantly increased in response to L-fuc. Additionally, a previous study reported that treatment with fucoidan, a sulfated polymer of L-fuc, can reduce *CCL22* mRNA transcription levels in macrophages (71), consistent with our findings (**Figure 3D**). Therefore, our findings support the hypothesis that free L-fuc, similar to fucoidan, interacts with DC-SIGN, activating a similar signaling downregulation of *CCL22* mRNA transcription levels in moDC. Further studies are warranted to elucidate this possibility.

Interestingly, while we observe an increase in abundance of both cDC1 and moDCs *ex vivo* (**Figure 1E**) and at early timepoints during our *in vivo* models (**Figure 4C**) at a late timepoint in our tumor model we observe a decrease in the moDC subset (**Figure 4D**). Our group has previously observed a similar finding in a melanoma tumor model (68). It is likely that this phenomenon is caused by intratumoral moDCs trafficking outside of the tumor to the lymph nodes. In the lymph node the moDCs then activate naïve T cells which will maintain the anti-tumor phenotype. Additionally, we observe that splenic DCs harvested 7 days after initiation of L-fuc increased T cell interferon-gamma (IFNγ) release regardless of whether the T cells received L-fuc treatment (**Figure 4E**). However, at 21 days after initiation of L-fuc, there is no difference in the amount of IFNγ produced in T cells from L-fuc-treated mice cultured with splenic DCs treated ± L-fuc (**Figure 4F**). Interestingly, at this timepoint the total level of IFNγ is higher in T cells harvest from L-fuc-treated mice cultured with splenic DCs not treated with L-fuc. This result may be attributed to increased DC-mediated activation of T cells induced by L-fuc in the spleens of the treated mice prior to their extraction for our ex vivo co-culture assays. Future studies are needed to understand the interaction of T cells and DCs after L-fuc treatment in order to elucidate how to leverage this mechanism for effective immunotherapies.

Our study establishes that L-fuc enhances antigen uptake and processing in DCs. Our data demonstrate that treatment of both bmDCs and splenic DCs results in increased antigen uptake, processing via lysosomal degradation of antigen, and antigen-loaded MHC ll as indicated by increased activation of antigen-specific T cells (**Figures 2A-F**, **4A, B, E**). Furthermore, our single-cell RNA sequencing (scRNAseq) data revealed that in cDC1s, L-fuc treatment stimulates upregulation of antigen-processing pathways and downregulations of negative regulators of antigen processing (**Figure 5B**) as early as 7 days after initiation of L-fuc treatment. Additionally, pathways associated with MHC l processing and presentation were also upregulated after L-fuc treatment suggesting that L-fuc treated DCs are more likely to cross-present antigen on MHC l and MHC ll complexes. Further studies are needed to elucidate the extent by which L-fuc-treated DCs are capable of cross-presentation. Findings in this regard would suggest a previously unidentified mechanism of cross-presentation regulation in DCs. While our group has previously shown that the MHC ll protein HLA-DRB1 is directly fucosylated and that this post-translational modification promotes its cell surface trafficking and accumulation to stimulate CD4^+^T cell-mediated anti-tumor immune responses, the fucosylation-mediated regulation of other MHC proteins on tumors and immune cells has yet to be established, and future studies are needed. Moreover, whether and how L-fuc and fucosylation may impact antigen peptide:MHC loading or alter initialization kinetics of the immunological synapse also remain unknown. Antigen decoration by glycosylation has previously been reported (104). However, to our knowledge, no previous study has delineated whether antigen fucosylation occurs and what potential immunological ramifications such a post-translational modification might cause. Given that MHC ll antigen loading has been described to occur in the vesicles (105) that originate from the endoplasmic reticulum and Golgi apparatus where fucosyltransferases reside (106), there is inherent subcellular co-localization of the peptide-loading process and fucosyltransferases that may make fucosylation of antigens possible. Thus, we hypothesize that internalized antigens may exhibit altered fucosylation profiles that stimulate enhanced T cell activity. Future studies are needed to elucidate antigen fucosylation and the effect that this may have on the immunological synapse.

Our findings that L-fuc promotes an enhanced immunostimulatory phenotype in DCs suggests a rationale to modulate forms of immunotherapy that primarily rely on DC. To this end, our findings support the concept of using L-fuc to enhance DCVs. Previous studies that have tested the efficacy of DCVs and found that vaccine products consisting of moDCs provided advantageous outcomes due to the ease of generation and functional ability to prime both a CD4^+^ and CD8^+^ T cell response (107, 108). As our findings reveal that L-fuc increases the abundance and immunostimulatory potential of moDCs, it is likely that L-fuc would enhance DCV efficacy. Additionally, our findings reveal that DCs derived from mice fed with L-fuc still exhibit increased antigen uptake and T cell priming capacity even after cessation of L-fuc treatment when the cells are cultured *ex vivo*. These findings suggest that the observed effects of L-fuc are sustained after cessation of treatment in the DCs. This implies that during the generation of DCV, DCs could be supplemented with L-fuc *ex vivo* and maintain a heightened immunostimulatory activity upon reinfusion into the patients without the need for further L-fuc treatment. However, further studies are needed to test how and when L-fuc can be administered to enhance the therapeutic efficacy of DCV product, and for how long the enhancement effects can be sustained after cessation of L-fuc treatment.

Although our *ex vivo* bmDC-based studies clearly demonstrate that L-fuc can enhance aspects of DC biology such as abundance, subtyping, and antigen processing, whether these effects are conserved or differ in tumor-derived DCs due to tumor microenvironmental interactions is unclear and is an ongoing focus of our group. However, given that the observed increases in DC abundance intratumorally and from BM-derived DCs are comparable, we are confident that the findings from bmDCs would translate to intratumor DCs. Additionally, bmDCs have previously been used to deduce specific aspects of DC biology using similar culturing conditions to that of this study (109–113), further supporting the validity of this methodology. Interestingly, through our *in vivo* and *ex vivo* studies we observed that L-fuc increases cDC1 and moDC abundance in similar proportions. Additionally, our findings that L-fuc increases the abundance of specific subsets of DCs and increases the ability of these DCs to prime T cell responses suggests that L-fuc has conserved effects on different DC subsets. Future studies are needed to mechanistically delineate how each individual subset of DCs might respond to L-fuc.

In conclusion, our study demonstrates the novel finding that L-fuc can modulate DC functionality to enhance immune responses. The enhanced functionality is associated with increased abundance of cDC1s and moDCs, upregulation of pathways associated with enhanced immunostimulatory activity and downregulation of pathways that suppress effector cell function. Future studies which seek to elucidate specific targetable genes in these cells are expected to enhance our understanding of not only L-fuc as an immunostimulatory molecule but also as novel adjuvant to be combined with immunotherapy, particularly for the enhancement of DCVs. Further studies are expected to provide insights that will inform the eventual strategic and rational application of L-fuc as a standard agent to enhance immunotherapy outcomes for cancer patients.

## Data availability statement

The data presented in the study are deposited in the Gene Expression Omnibus (GEO) repository, accession numbers GSE261035 and GSE261971.

## Ethics statement

The animal study was approved by Institutional Animal Care and Use Committee (IACUC) of University of South Florida and Moffitt Cancer Center. The study was conducted in accordance with the local legislation and institutional requirements.

## Author contributions

CB: Conceptualization, Data curation, Formal analysis, Methodology, Resources, Supervision, Validation, Visualization, Writing – original draft, Writing – review & editing. AB: Data curation, Validation, Writing – review & editing. KS: Data curation, Writing – review & editing. CZ: Data curation, Writing – review & editing. SY: Data curation, Methodology, Writing – review & editing. DD: Data curation, Formal analysis, Writing – review & editing. DA: Resources, Writing – review & editing. XY: Data curation, Formal analysis, Methodology, Writing – review & editing. EL: Conceptualization, Funding acquisition, Methodology, Resources, Supervision, Visualization, Writing – review & editing, Writing – original draft.

## Supplementary material

Supplementary Table 1 Real-time PCR primers.

Supplementary Table 2 cDC1 single cell RNA sequencing gene list.

Supplementary Figure 1 Flow cytometry gating strategies. (1) Cells were first gated by forward and side scatter to remove debris. (2) CD45^+^ cells were isolated with living cells to remove low viability and non-immune cells. (3) Total DCs were isolated from non-myeloid and macrophages as CD11c^+^F4/80^-^. (4) Subsets of DCs were separated into cDC1 and non-cDC1 by the presence of CD11b. (5) cDC2s and moDCs were separated by the presence of CD103 and Ly6C as indicated.

Supplementary Figure 2 Dose titration response of L-fucose in breast cancer. Growth curves of TUBO tumors in mice fed the indicated dosage of L-fuc (n = 5 mice per cohort, error presented as SEM).

Supplementary Figure 3 Macrophage abundance from bmDC maturation. Flow cytometric analysis of CD45^+^F4/80^+^ macrophages following bmDC treatment ± L-fuc for 6 days (representative figure of 3 replicates, n = 3, error presented as SEM).

Supplementary Figure 4 Major histocompatibility complex ll expression on dendritic cell subtypes after L-fucose treatment. **(A)** Flow cytometric analysis of MHC^+^ DC subtypes in bmDCs treated ± L-fuc for 6 days prior to incubation with DQ-OVA for 1 hour (representative figure of 3 replicates, n = 4, error presented as SEM). **(B)** Flow cytometric analysis of MHC ll abundance per cell on each DC subtype reported as MFI (representative figure of 3 replicates, n = 4, * = p > 0.05, error presented as SEM).

Supplementary Figure 5 Treatment Schematic for bmDC timecourse.

Supplementary Figure 6 Single-cell RNA sequencing identifies immune subsets altered after L-fuc treatment. **(A)** UMAP representing identified CD45^+^ populations clusters using relevant gene markers. **(B)** UMAPs displaying identified CD45^+^ clusters correlating to identified treatment and timepoints. **(C)** Gene expression sets used to identify individual CD45^+^ clusters as previously shown. **(D)** Volcano plot displaying changes in gene expression in the cDC1 cluster between day 7 and day 0 of control- and L-fuc-treated tumors.
