# Supplementary figures and images for: The functional role of L-fucose on dendritic cell function and polarization
Source: Front Immunol. 2024 Apr 5;15:1353570. doi: 10.3389/fimmu.2024.1353570 (PMC11026564; doi:10.3389/fimmu.2024.1353570)

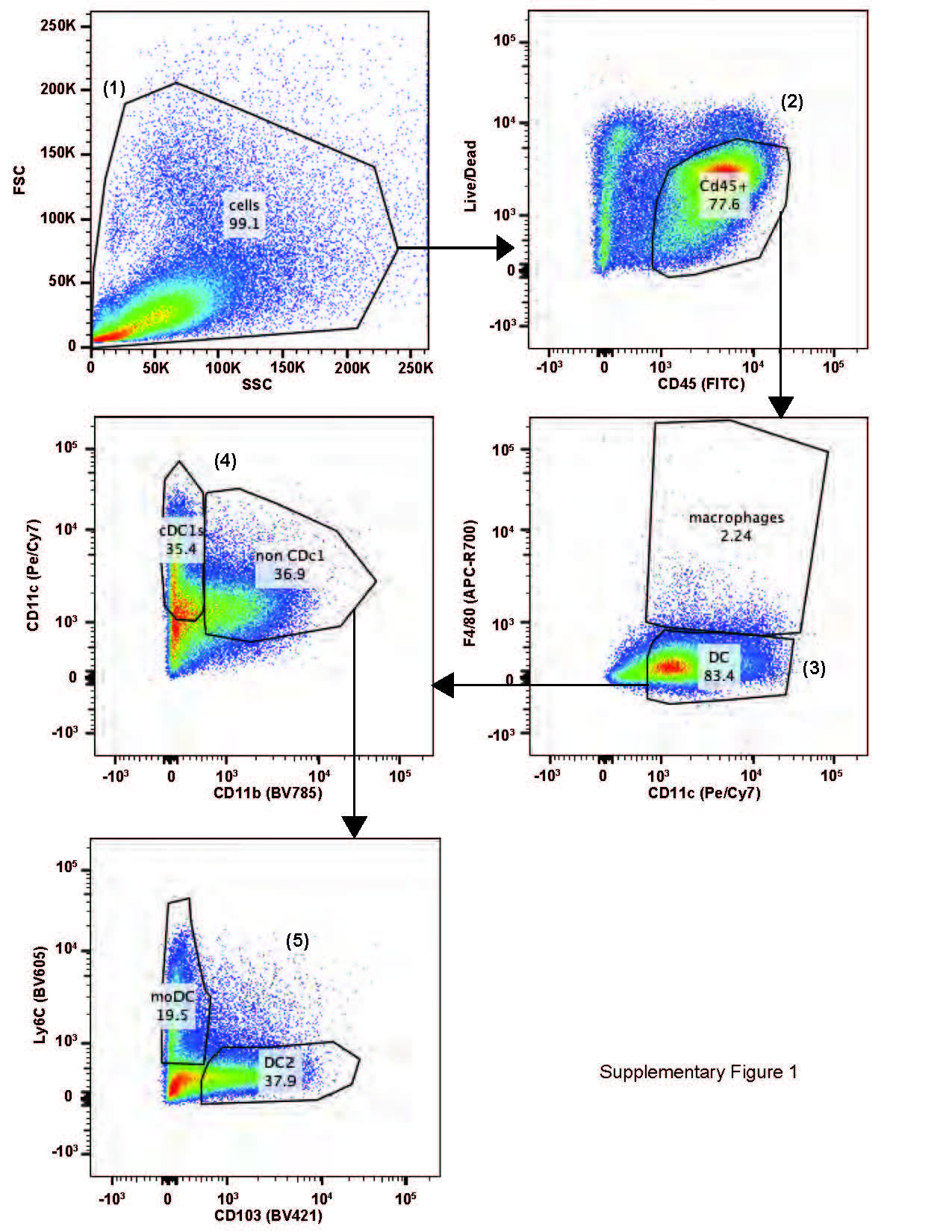

Supplement: Supplementary Figure 1 — Flow cytometry gating strategies. (1) Cells were first gated by forward and side scatter to remove debris. (2) CD45+ cells were isolated with living cells to remove low viability and non-immune cells. (3) Total DCs were isolated from non-myeloid and macrophages as CD11c+F4/80-. (4) Subsets of DCs were separated into cDC1 and non-cDC1 by the presence of CD11b. (5) cDC2s and moDCs were separated by the presence of CD103 and Ly6C as indicated. [file Image_1.jpeg]

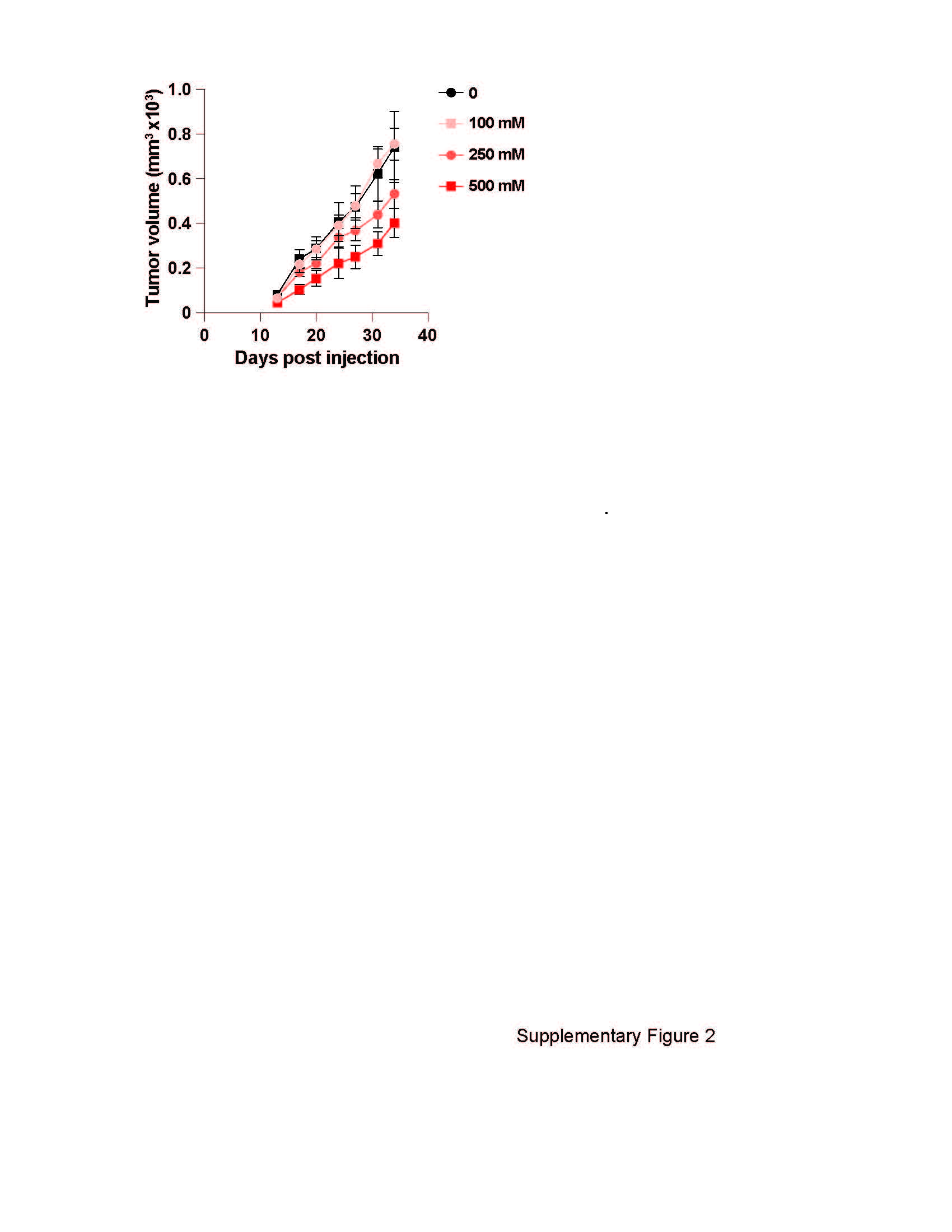

Supplement: Supplementary Figure 2 — Dose titration response of L-fucose in breast cancer. Growth curves of TUBO tumors in mice fed the indicated dosage of L-fuc (n = 5 mice per cohort, error presented as SEM). [file Image_2.jpeg]

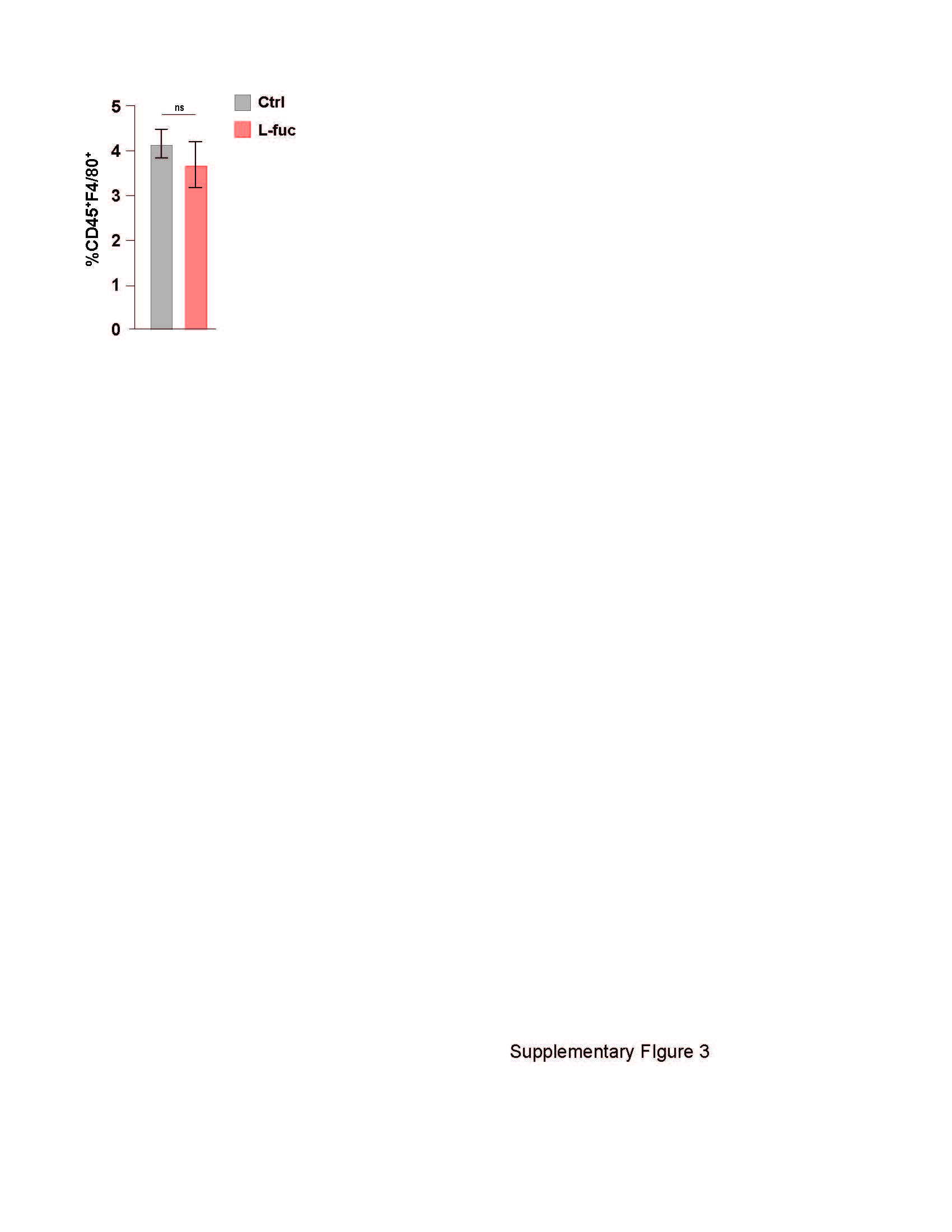

Supplement: Supplementary Figure 3 — Macrophage abundance from bmDC maturation. Flow cytometric analysis of CD45+F4/80+ macrophages following bmDC treatment ± L-fuc for 6 days (representative figure of 3 replicates, n = 3, error presented as SEM). [file Image_3.jpeg]

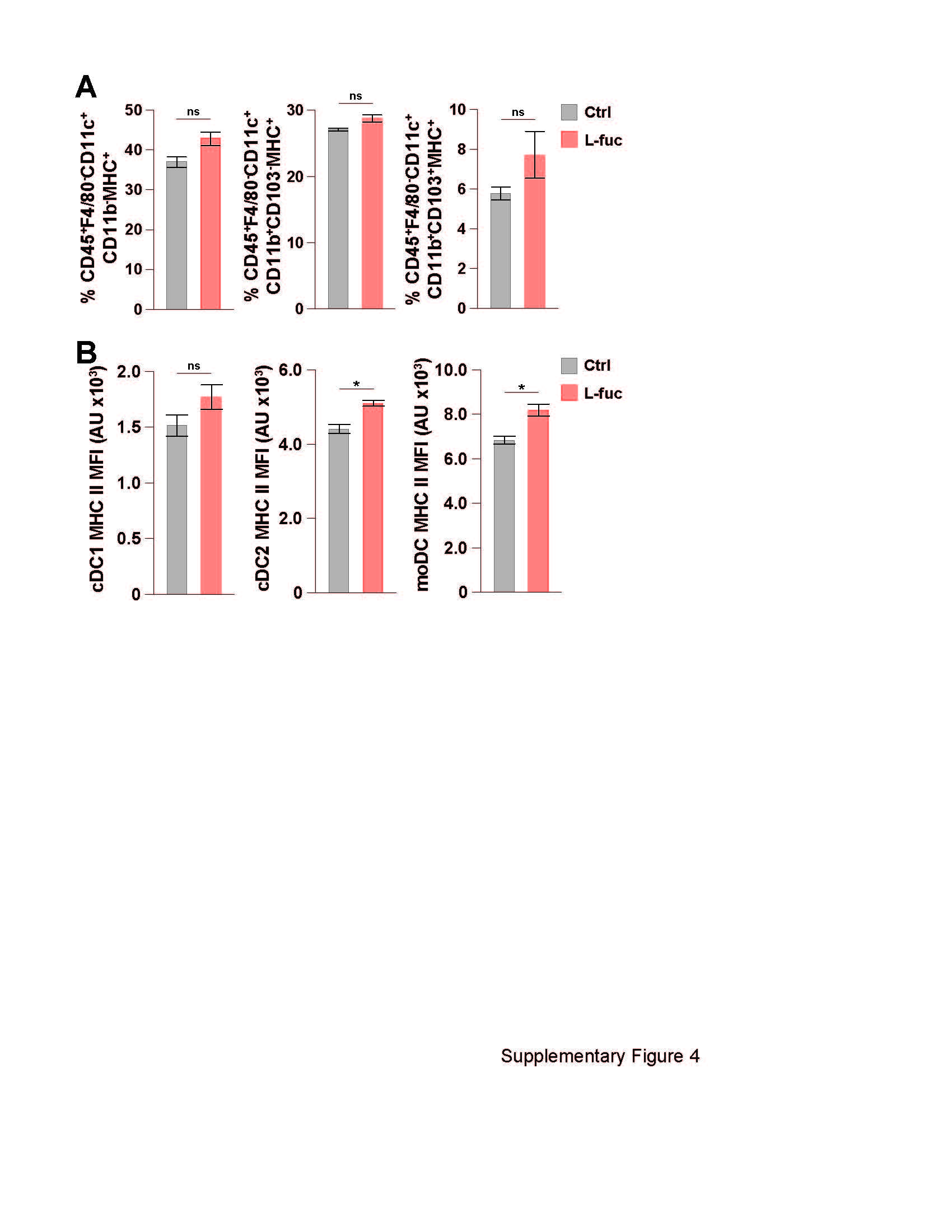

Supplement: Supplementary Figure 4 — Major histocompatibility complex ll expression on dendritic cell subtypes after L-fucose treatment. (A) Flow cytometric analysis of MHC+ DC subtypes in bmDCs treated ± L-fuc for 6 days prior to incubation with DQ-OVA for 1 hour (representative figure of 3 replicates, n = 4, error presented as SEM). (B) Flow cytometric analysis of MHC ll abundance per cell on each DC subtype reported as MFI (representative figure of 3 replicates, n = 4, * = p > 0.05, error presented as SEM). [file Image_4.jpeg]

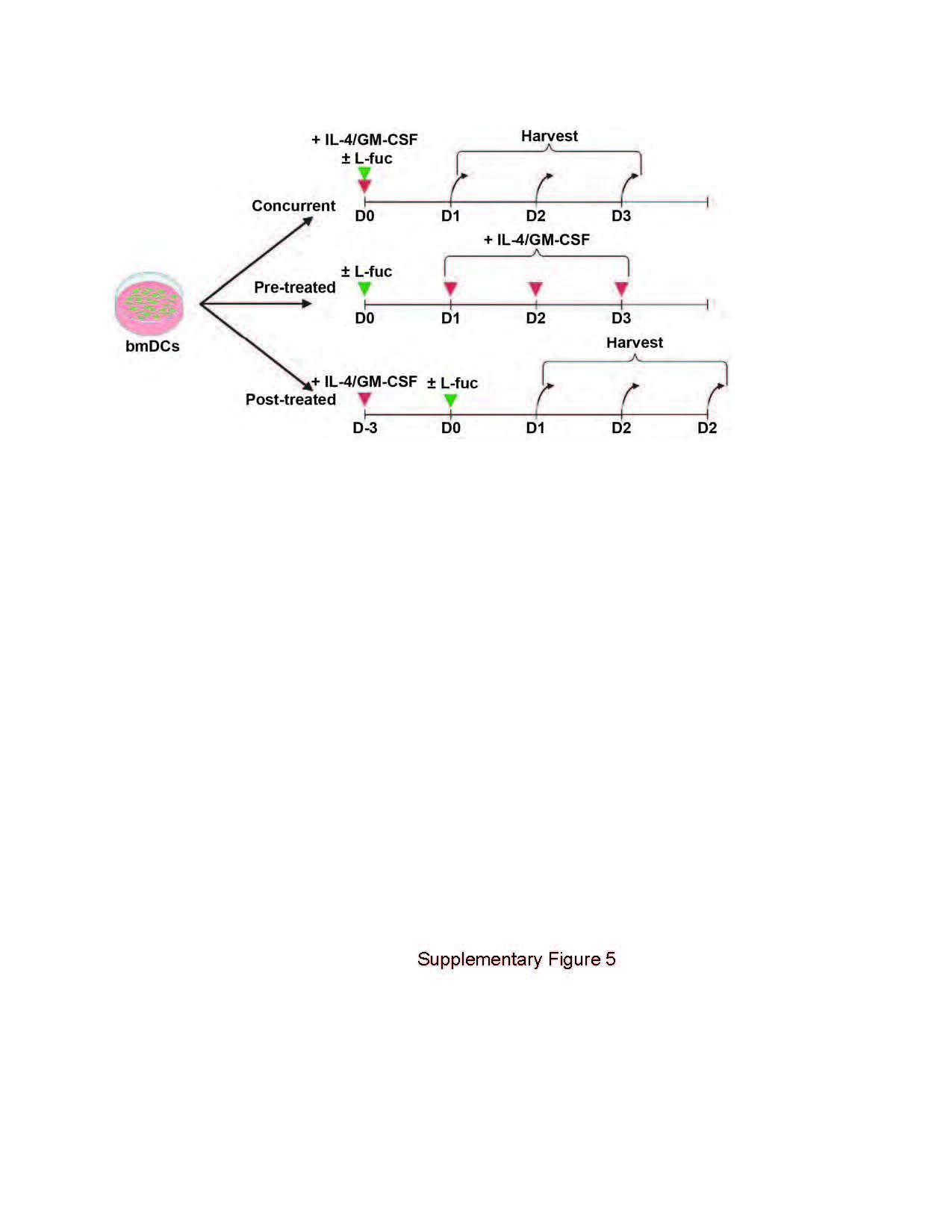

Supplement: Supplementary Figure 5 — Treatment Schematic for bmDC timecourse. [file Image_5.jpeg]

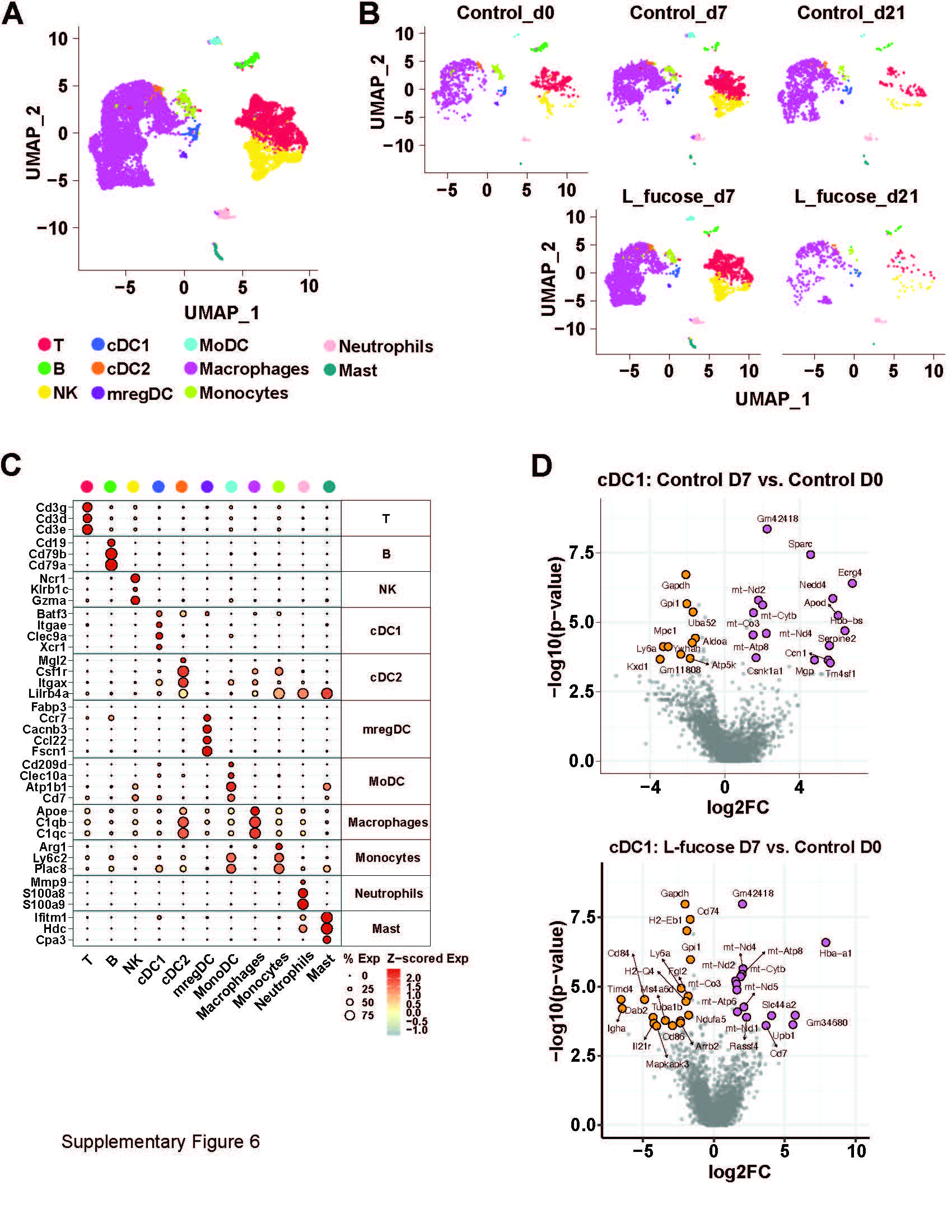

Supplement: Supplementary Figure 6 — Single-cell RNA sequencing identifies immune subsets altered after L-fuc treatment. (A) UMAP representing identified CD45+ populations clusters using relevant gene markers. (B) UMAPs displaying identified CD45+ clusters correlating to identified treatment and timepoints. (C) Gene expression sets used to identify individual CD45+ clusters as previously shown. (D) Volcano plot displaying changes in gene expression in the cDC1 cluster between day 7 and day 0 of control- and L-fuc-treated tumors. [file Image_6.jpeg]
